# Sex differences in the neuroadaptations associated with incubated cocaine-craving: A focus on the dorsomedial prefrontal cortex

**DOI:** 10.3389/fnbeh.2022.1027310

**Published:** 2023-01-05

**Authors:** Eleanor Blair Towers, Madison Kilgore, Anousheh Bakhti-Suroosh, Lasyapriya Pidaparthi, Ivy L. Williams, Jean M. Abel, Wendy J. Lynch

**Affiliations:** ^1^Psychiatry and Neurobehavioral Sciences, University of Virginia, Charlottesville, VA, United States; ^2^Medical Scientist Training Program, University of Virginia, Charlottesville, VA, United States

**Keywords:** cocaine-craving, sex differences, telescoping effect, dmPFC, *Bdnf-IV*, *Grin2a*, *Grin2b*, *Grin1*

## Abstract

**Introduction:**

Women have a shorter course from initial cocaine use to meeting the criteria for cocaine use disorder as compared to men. Preclinical findings similarly indicate that females develop key features of an addiction-like phenotype faster than males, including an enhanced motivation for cocaine and compulsive use, indicating that this phenomenon is biologically based. The goals of this study were to determine whether cocaine-craving, another key feature of addiction, also develops sooner during withdrawal in females than males and to determine whether there are sex differences in the molecular mechanisms associated with its development focusing on markers known to mediate cocaine-craving in males (i.e., dorsomedial prefrontal cortex, dmPFC, expression of brain-derived neurotrophic factor exon-IV, *Bdnf-IV*, and NMDA receptor subunits, *Grin2a, Grin2b*, and *Grin1*).

**Methods:**

Cocaine-craving was assessed following extended-access cocaine self-administration and 2, 7, or 14 days of withdrawal using an extinction/cue-induced reinstatement procedure. Tissue was obtained from the dmPFC immediately after reinstatement testing and gene expression changes were analyzed using real-time qPCR.

**Results:**

In males, cocaine-craving (total extinction and cue-induced reinstatement responding) progressively increased from early to later withdrawal time-points whereas in females, cocaine-craving was already elevated during early withdrawal (after 2 days) and did not further increase at later withdrawal time-points. Levels of cocaine-craving, however, were similar between the sexes. Gene expression changes differed markedly between the sexes such that males showed the expected relapse- and withdrawal-associated changes in *Bdnf-IV, Grin2a, Grin2b*, and *Grin1* expression, but females only showed a modest increase *Grin1* expression at the intermediate withdrawal timepoint.

**Discussion:**

These findings indicate that cocaine-craving is similarly expressed in males and females although the time-course for its incubation appears to be accelerated in females; the molecular mechanisms also likely differ in females versus males.

## 1. Introduction

Overdose deaths involving cocaine have been steadily increasing since 2014 (CDC WONDER, 2022). Despite men having higher rates of cocaine use disorder than women (Substance Abuse and Mental Health Services Administration [SAMHSA], 2020), woman are more vulnerable to many aspects of the disease. For example, women have a shorter time period from initial cocaine use to meeting the criteria for cocaine use disorder and/or seeking treatment for the disorder as compared to men (Griffin et al., 1989; White et al., 1996; McCance-Katz et al., 1999; Sofuoglu et al., 1999; Haas and Peters, 2000; Hernandez-Avila et al., 2004; O’Brien and Anthony, 2005). This phenomenon has been termed the “telescoping effect” and is a consistent effect that has been reported across multiple drug classes including psychostimulants, such as cocaine and methamphetamine (Griffin et al., 1989; White et al., 1996; McCance-Katz et al., 1999; Sofuoglu et al., 1999; Haas and Peters, 2000; Brecht et al., 2004; Hernandez-Avila et al., 2004; O’Brien and Anthony, 2005) as well as other addictive drugs, including alcohol, opioid, tobacco, and cannabis (Anglin et al., 1987; Hser et al., 1987a,b; Haas and Peters, 2000; DiFranza et al., 2002; Hernandez-Avila et al., 2004; Ehlers et al., 2010; Back et al., 2011a,b; Khan et al., 2013; Lewis et al., 2014; Adelson et al., 2018; Peltier et al., 2021) and non-pharmacological addictions, such as gambling (Ladd and Petry, 2002; Ibanez et al., 2003; Tavares et al., 2003; Grant et al., 2012). Women with a cocaine use disorder also experience more drug-related medical and psychological complications, report greater stress-induced cravings, and longer periods of use after relapse compared to their male counterparts (White et al., 1996; Luchansky et al., 2000; Gallop et al., 2007; Center for Substance Abuse Treatment, 2009; Greenfield et al., 2010; Potenza et al., 2012; Becker and Koob, 2016).

While this increased risk in women may be due to socio-cultural factors (Becker et al., 2016), results from preclinical studies indicate that biological factors also contribute given that female animals develop addiction-like features more readily than male animals (Lynch and Taylor, 2004; Kerstetter et al., 2012; Perry et al., 2013, 2015; Kawa and Robinson, 2019; Towers et al., 2021). For example, female rats self-administer higher levels of cocaine, show greater escalation of cocaine intake over time, and have a more significant disruption of diurnal control over drug intake than males under extended-access cocaine self-administration conditions, which supports an enhanced vulnerability to transition from controlled to dysregulated cocaine use in females (Lynch and Taylor, 2004, 2005; Roth and Carroll, 2004; Smith et al., 2011; Kawa and Robinson, 2019; Algallal et al., 2020). Additionally, our more recent study showed that two addiction-like features, an enhanced motivation for cocaine and compulsive cocaine use despite negative consequences, developed sooner during withdrawal from extended-access cocaine self-administration in females compared to males (following 7 versus 14 days; Towers et al., 2021). Our previous study and work from others has also shown that these addiction-like features increase, rather than decrease, in magnitude over withdrawal (Gancarz-Kausch et al., 2014; Towers et al., 2021). This incubation effect has been extensively described for cue-induced cocaine-craving, which increases progressively, or incubates, over protracted withdrawal in both humans and animals tested following extended-access drug self-administration (Grimm et al., 2001; Li et al., 2015). This incubation effect also occurs in both males and females (Nicolas et al., 2022) and is particularly robust in females tested during estrus (versus non-estrus phases and males; Nicolas et al., 2019; Corbett et al., 2021). However, it remains unknown whether there are sex differences in the time-course for the incubation of cue-induced cocaine-craving.

The molecular mechanisms underlying incubated cocaine-craving are also largely unknown in females since the vast majority of the work has been conducted in male animals only. Determining sex differences in the underlying neuroadaptations of key features of cocaine use disorder, such as the incubation of cocaine-craving, will be imperative as the effort to develop the first FDA-approved treatment for cocaine use disorder continues. While not yet examined, glutamatergic signaling is a strong candidate as a mechanism underlying the telescoping effect given the critical role this pathway plays in mediating drug-craving/relapse in both humans and animal models (Rebec and Sun, 2005; Goldstein and Volkow, 2011; Kalivas and Volkow, 2011; Szumlinski and Shin, 2018). Findings from male animals further indicates a causal role for glutamate receptor signaling in the dorsomedial prefrontal cortex (dmPFC) in drug-craving and its incubation over withdrawal (McClure et al., 2014; Szumlinski and Shin, 2018). Specifically, glutamatergic signaling in this pathway changes dramatically during withdrawal, from hypoglutamatergic during early withdrawal, when levels of drug-craving are low, to hyperglutamatergic during late withdrawal, when craving has increased to high levels (after 7 or more days; Ben-Shahar et al., 2009, 2012, 2013; Chen et al., 2013; Sun et al., 2014; Funk et al., 2016; Koob and Volkow, 2016; Barry and McGinty, 2017; Hearing et al., 2018; Szumlinski and Shin, 2018; Siemsen et al., 2019; Caffino et al., 2020; Roura-Martínez et al., 2020). NMDA receptors have been shown to be critically involved in the early-withdrawal molecular cascade that triggers the incubation of craving (Barry and McGinty, 2017), as well as the enhanced cue-induced craving following late withdrawal (Chen et al., 2013; Barry and McGinty, 2017; Szumlinski and Shin, 2018). Brain derived neurotropic factor (BDNF) signaling, which requires coincident activation by glutamate and dopamine, also increases in the dmPFC following relapse testing (Peterson A. et al., 2014; Abel et al., 2019) and clinical studies have similarly shown elevated BDNF serum levels are predictive of cocaine craving and vulnerability to relapse in abstinent cocaine-dependent individuals (D’Sa et al., 2011; Corominas-Roso et al., 2013). Although glutamatergic regulation of cocaine-craving has not yet been examined in females, recent findings with methamphetamine show that extended-access self-administration differently impacts excitatory signaling in the PFC of females versus males (Pena-Bravo et al., 2019). Further support is provided by data showing that estradiol induces glutamate release (Le Saux et al., 2006), mediates synaptic plasticity in brain through a glutamate-dependent mechanism (Peterson B. et al., 2014), and earlier data showing sex-specific differences in NMDA receptor functioning (Cyr et al., 2001).

The purpose of the present study was to expand on our previous study on the telescoping effect and determine whether cocaine-craving, as assessed using an extinction/cue-induced reinstatement procedure, also incubates sooner during withdrawal in females than males. We also determined whether the molecular mechanisms associated with incubated cocaine-craving differed between males and females and from early to later periods of withdrawal focusing on markers known to mediate cue-induced cocaine-craving in males (brain-derived neurotrophic factor exon-IV, *Bdnf-IV*, and NMDA receptor subunit-related gene expression, *Grin1*, *Grin2a*, *Grin2b*, in the dorsomedial prefrontal cortex, dmPFC; Peterson A. et al., 2014; Szumlinski et al., 2016; Abel et al., 2019). Based on previous findings indicating that addiction-like features, including cocaine-craving, develop following extended-access self-administration and increase, or incubate, over withdrawal (Grimm et al., 2001; Gancarz-Kausch et al., 2014; Towers et al., 2021), effects were examined during early withdrawal (following 2 days; W2), which was expected to be sub-threshold for inducing an addiction-like phenotype, intermediate withdrawal (following 7 days; W7), which was expected to be threshold for inducing an addiction-like phenotype, and late withdrawal (following 14 days; W14), which was expected to be optimal for inducing an addiction-like phenotype. Given our previous findings (Towers et al., 2021) and reports of a telescoping effect in women, we hypothesized that cocaine-craving (extinction and cue-induced reinstatement) would incubate to high levels sooner during withdrawal in females versus males. Additionally, we predicted that BDNF and NMDA receptor gene expression in the dmPFC would correspond to withdrawal- and sex-dependent differences in cocaine-craving.

## 2. Materials and methods

### 2.1. Subjects

Sexually mature female (*N* = 52) and male (*N* = 41) Sprague–Dawley rats (Charles River) were used as subjects in this study. Rats were approximately 11-weeks-old and weighed roughly 260 g (females) and 380 g (males) at the start of the study. Upon arrival at the facility, rats were individually housed in operant conditioning chambers (Med Associates Inc., St. Albans, VT, USA) with *ad libitum* access to water and food (Teklad LM-485 7912; except as noted below for some animals during cocaine self-administration training) and maintained on a 12-h light/dark cycle (lights on at 7 a.m.). Over the course of the study, the rats were weighed three times a week and their health were monitored daily. All procedures were conducted within animal care guidelines set by the National Institution of Health and were approved by The University of Virginia Animal Care and Use Committee.

### 2.2. Procedures

#### 2.2.1. Lever pre-training

Following a 2-day habituation period to the operant chamber, rats were pre-trained to lever press for sucrose pellets (45 mg) in order to ensure rapid subsequent acquisition of cocaine self-administration using methods previously described (Lynch, 2008; fixed-ratio 1 access, 24-h/day). Briefly, pellets were available 24h/day under a fixed ratio 1 (FR1) schedule; no stimulus was paired with sucrose pellet delivery. Acquisition was defined as 2 consecutive days wherein >50 pellets were obtained. Rates of acquisition of lever-pretraining did not differ between males and females (days ± SEM, 2 ± 0.06 versus 2 ± 0.11, respectively).

#### 2.2.2. Surgery and catheter maintenance

After lever pre-training, an indwelling catheter (Silastic tubing; 0.51 and 0.94 mm o.d.; Dow Corning, Midland, MI, USA) was implanted into the right jugular vein of the rats using methods previously described (Lynch, 2008). Catheter patency was maintained and verified throughout the study by flushing with heparinized saline 3 days/week. If the patency of a catheter was questionable, it was verified by administering sodium brevital (1.5 mg/kg). Any catheter that was no longer patent (i.e., the catheter was leaking, pressure prevented flushing, or the animal did not lose the righting reflex immediately after sodium brevital) was replaced by a new catheter that was implanted into the left jugular vein with testing resuming following recovery from surgery (1–2-days). Data collected between this assessment and the last patency check were discarded.

#### 2.2.3. Cocaine self-administration training

**FIGURE 1:**
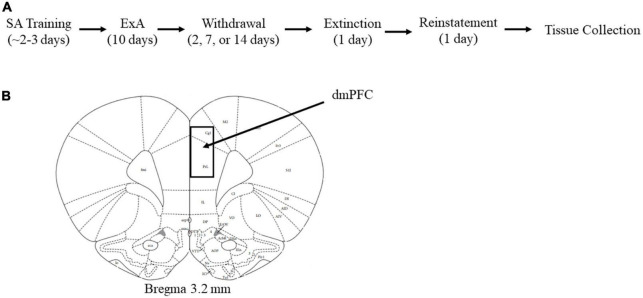
Summary of experimental events and brain region dissected for the gene expression analyses. **(A)** Timeline of experimental events: Following self-administration training (SA), rats were given 24-h/day extended (ExA) access to cocaine (1.5 mg/kg/infusion) under a discrete trial procedure for 10 days (4 trials/h). After the last cocaine self-administration session, rats were assigned to an early (*n* = 13 females, 10 males), intermediate (*n* = 13 females, 10 males), or late (*n* = 13 females, 10 males) withdrawal groups. Additional rats were given access to saline and assigned to an early (*n* = 4 females, 5 males), intermediate (*n* = 4 females, 4 males), or late (*n* = 6 females, 2 males) withdrawal groups. Following the appropriate days of withdrawal, rats underwent extinction testing in a minimum of 6, 1-h sessions and then the next day reinstatement testing in a 1-h session. Tissue was collected immediately after the reinstatement session. **(B)** Schematic illustration of the brain region dissected for the dorsomedial prefrontal cortex (dmPFC).

Following surgery, rats were trained to self-administer cocaine (Figure 1A; 1.5 mg/kg/infusion, a relatively high dose shown to encourage rapid rats of acquisition) under a FR1 schedule with a maximum of 20 infusions/day using methods previously described (Lynch et al., 2010). Each session began with the introduction of the active lever (cocaine-associated lever) into the left side of the operant chamber and remained extended until all 20 infusions were obtained or until the session ended at 11:50 a.m. the next day. Responses on the active lever were reinforced with an infusion of cocaine under a fixed-ratio 1 schedule; infusions were paired with the sound of the infusion pump located inside the sound attenuated chamber and a stimulus light located above the active lever. The active lever retracted following the 20th infusion (or at 11:50 a.m.) and remained retracted until the next session. Responses on the non-active (right) lever were counted during the session to measure general activity, but did not have any programmed consequence. Acquisition was defined as two consecutive days wherein 20 infusions were obtained. Moderate food restriction (85% of its free-feeding body weight, roughly 20 g/day and 15 g/day for males and females, respectively) was used briefly (2–3 days) when necessary (i.e., fewer than 15 infusions/day by training day 5; one male in the W2 group, one male and one female in the W7 group, and two males in the W14 group). All groups acquired cocaine self-administration rapidly under these high dose conditions (typically within 2–3 days) and rates of acquisition did not differ between males and females or withdrawal groups.

#### 2.2.4. Extended-access cocaine self-administration and withdrawal

Once rats acquired cocaine self-administration, they were given 24-h/day, intermittent-access to cocaine (1.5 mg/kg/infusion) under a discrete trial procedure using methods previously described (Ramôa et al., 2013, 2014). Briefly, there were 4, 10-min discrete trials each hour that began every 15 min (up to 96 infusions/day) with the extension of the active-lever into the chamber. Cocaine was available during each of the trials under a fixed-ratio 1 schedule; each infusion was paired with the sound of the infusion pump and a stimulus light located above the active lever. Trials terminated, and the lever retracted, after an infusion was obtained or after 10 min. The inactive (right) lever remained extended the entire duration of the session and responses on the lever were recorded but had no consequence. Sessions ran continuously for 10 days. These extended-, intermittent-access conditions have been shown to induce ‘binge/abstinent’ patterns of intake and lead to the development an addiction-like phenotype when assessed following protracted withdrawal (Ramôa et al., 2013; Towers et al., 2021). After the last extended-access session, cocaine was again available for two additional sessions under fixed-ratio 1 session (up to 20 infusions) in order to equate intake between the groups prior to withdrawal. Based on our previous findings showing that females develop an addiction-like phenotype sooner during withdrawal than males (7 versus 14 days; Towers et al., 2021), we assigned males and females randomly to one of three different withdrawal conditions: early (2 days; *n* = 14 and 10, respectively), intermediate (7 days; *n* = 14 and 10, respectively), or late (14 days; *n* = 14 and 11, respectively) withdrawal. The withdrawal period began following the second FR1 session. Rats remained in their operant chambers during withdrawal. Additional male and female rats were also randomly assigned to an early (*n* = 4 and 5, respectively), intermediate (*n* = 4 and 4, respectively), or late (*n* = 6 and 2, respectively) withdrawal groups; they underwent the same procedures as those described for the rats given extended-access to cocaine except they had access to saline instead of cocaine.

#### 2.2.5. Extinction/reinstatement testing

Extinction/reinstatement testing was conducted in two consecutive sessions after 2, 7, or 14 days of withdrawal. First, extinction responding was examined in a minimum of 6, 1-h sessions using methods previously described (Peterson A. et al., 2014; Sanchez et al., 2014; Beiter et al., 2016). These sessions started between 9 and 10:00 a.m. with the re-introduction of the active-lever into the operant chamber; responses on this lever and the inactive lever were recorded but had no programmed consequence. Sessions continued until responding on the active lever met an extinction criterion of less than 15 responses/h. This criterion was typically met within 6 sessions and all rats extinguished within 8 sessions. Cue-induced reinstatement responding was assessed the following day in a 1-h session. This session also started between 9 and 10:00 a.m. with the re-introduction of the active lever into the operant chamber as well as a presentation of the cues formerly associated with cocaine (sound of infusion pump and the light above the active-lever; roughly 3–5 s based on body weight). Each response on the left-lever produced these same cues. Female rats were also swabbed 3 days prior to the extinction and reinstatement test days and on the behavioral test days as described previously (Lynch et al., 2019) to determine the phase of the estrous cycle. Male rats underwent similar handling. Unfortunately, the phase of the estrous cycle could not be determined because the slides were contaminated during staining and unable to be interpreted. Therefore, the phase of the estrous cycle was not included in the data analysis. Additionally, one female in the early withdrawal group, one female in the intermediate withdrawal group and one female and one male in the late withdrawal group were excluded from the study and all analyses due to patency issues during extended-access self-administration or technical issues during withdrawal/relapse testing.

The final group size for females and males in the cocaine groups was 13 and 10 for the early withdrawal group, 13 and 10 for the intermediate withdrawal group, and 12 and 10 for the late withdrawal group. For the saline groups, since there were no behavioral differences between rats tested following early, intermediate, or late withdrawal, data were collapsed into one group (*n* = 14 females and 11 males).

#### 2.2.6. Gene expression

Immediately following the reinstatement session, rats were anesthetized using isoflurane and then euthanized by rapid decapitation. Tissue from the dmPFC was rapidly dissected from ice-chilled 2-mm-thick coronal brain slices based on boundaries defined previously (Figure 1B; coordinates: Bregma 3.2 mm; Paxinos and Watson, 2007). The dmPFC, which includes the prelimbic region and anterior cingulate, was selected since this region is known to be critical for the incubation of cocaine-craving in males (Kalivas and McFarland, 2003; Ishikawa et al., 2008). The tissue was immediately frozen using liquid nitrogen and stored at −80°C until further processing.

RNA extraction, cDNA transcription, and RT-qPCR were performed using methods previously described (Smith et al., 2018). Briefly, total RNA was isolated using a RNeasy (R) Lipid Tissue Mini Kit (Qiagen, Valencia, CA, USA) and the quantity and quality was determined using a NanoDrop™ Spectrophotometer. cDNA templates were prepared using High-Capacity cDNA Reverse Transcription Kit with RNase Inhibitor (Applied Biosystems, Carlsbad, CA, USA). RT-qPCR was performed using the Applied Biosystem StepOnePlus™ real-time PCR system and Applied Biosystems TaqMan™ Gene Expression assays using oligonucleotide primers chosen from prior publications. We analyzed the relative expression *of Bdnf-IV*, *Grin1*, *Grin2a*, and *Grin2b* (Supplementary Table 1). One housekeeping gene (*Gapdh* for Bdnf-associated genes or *B2m* for glutamate genes) was used for normalization. Since multiple qPCR plates were required for a plate study analysis for each gene, males and females, matched for group, were run on the same plate to eliminate potential sex differences due to qPCR plate run. A small number of samples were outliers and excluded based on the Grubb’s test, which included 8 of the 320 samples. The final group sizes of females and males for *Bdnf-IV* were 13 and 9 for the early withdrawal group, 12 and 10 for the intermediate withdrawal group, 12 and 10 for the late withdrawal group, and 14 and 11 for the saline controls, for *Grin1* were 13 and 10 for the early withdrawal group, 13 and 10 for the intermediate withdrawal group, and 12 and 10 for the late withdrawal group and 14 and 10 for the saline controls, for *Grin2a* were 13 and 9 for the early withdrawal group, 13 and 9 for the intermediate withdrawal group, and 12 and 9 for the late withdrawal group and 14 and 11 for the saline controls, and for *Grin2b* were 13 and 9 for the early withdrawal group, 13 and 10 for the intermediate withdrawal group, and 12 and 9 for the late withdrawal group and 14 and 11 for the saline controls.

### 2.3. Drugs

Cocaine hydrochloride was obtained from the National Institute on Drug Abuse (Research Triangle Park, NC, USA) and dissolved in 0.9% sterile saline (7 mg/ml), filtered (0.22 μm; Millipore, Billerica, MA, USA), and stored at 4°C. The infusion duration was adjusted three times/week based on body weight to maintain a constant mg/kg dose (2 s/100 g).

### 2.4. Data analysis

Data were first examined to verify that the assumptions of parametric analyses were met (i.e., normal distribution, equal variances). Sex differences were examined using both between sex analyses, to address quantitative differences, and using separate analyses within each sex, to address qualitative sex differences. This approach is analogous to the one we used recently to determine sex differences in the development of other addiction-like features (Towers et al., 2021) and has been recommended by other experts in the addiction field for detecting quantitative versus qualitative sex differences (Beltz et al., 2019). Specifically, quantitative differences in cocaine intake over the 10-day extended-access period were addressed using a repeated measures ANOVA with session as the repeated measure and sex and withdrawal group as the between-subject factors. *Post hoc* comparisons of the first and later sessions (averaged across sessions 2–10) were made using the paired *t*-test. Sex and withdrawal group were also included as between subject factors in the analyses of total extinction responses (univariant ANOVA), extinction responses during the first six extinction sessions run (repeated measures ANOVA), and responses during the last extinction session versus the reinstatement session (repeated measures ANOVA). *Post hoc* comparison of the first extinction session versus later sessions (averaged across sessions 2–6) and of the last extinction session versus the reinstatement session were made using the paired *t*-test. Withdrawal-dependent changes in extinction and reinstatement responding were also examined within females and males separately to address expected qualitative differences (e.g., ovarian hormones contribute to cocaine-craving in females, but not males; Jackson et al., 2006; Becker, 2009). Pearson correlations were also conducted to determine associations between average cocaine intake during extended-access and the total number of extinction and cue-induced reinstatement responses.

Similar analyses were used to determine quantitative and qualitative sex differences in gene expression with separate univariate ANOVA analyses conducted for each gene. Sex and withdrawal group differences were determined using percent difference from saline controls. Changes from baseline/saline expression levels were determined using a one-sample *t*-test (versus no change), and one-tailed *t*-tests were used for all *a priori* predicted hypotheses (i.e., higher cocaine-craving and *Bdnf-IV* and *Grin1* expression in the W7 and W14 withdrawal groups compared to the W2 withdrawal group*;* Abel et al., 2019). Partial eta squared (η_*P*_^2^) was used as a measure of effect size and Tukey correction was used to control for multiple comparisons. Statistical analyses were performed using SPSS (V26).

## 3. Results

### 3.1. Behavioral results

#### 3.1.1. Extended-access self-administration

**FIGURE 2:**
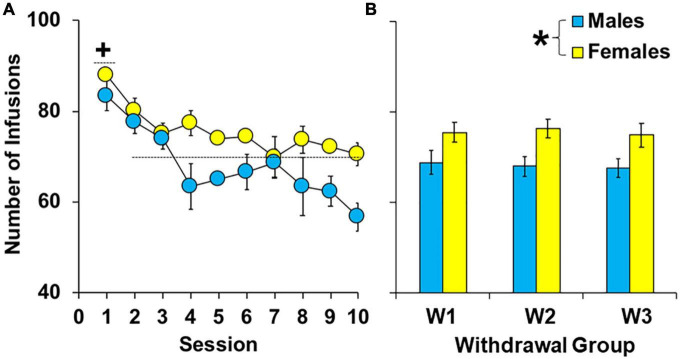
Effect of sex on cocaine intake during 10 extended-access sessions under a discrete trial procedure. Mean (±SEM) number of infusions obtained during each of the ten extended-access sessions **(A)** and averaged across all extended-access sessions **(B)** for males and females in the early (W2; *n* = 13 females, 10 males), intermediate (W7; *n* = 13 females, 10 males), and late (W14; *n* = 13 females, 10 males) withdrawal groups. *Significantly higher cocaine intake in females compared to males. + Significantly higher cocaine intake during the first extended-access session compared to later ones (averaged across sessions 2–10) for both sexes.

During the 10-day extended-access period, female rats self-administered more cocaine than males [Figures 2A, B; effect of sex, *F*_(1,62)_ = 15.16, *P* < 0.001]. However, there were no overall or interactive effects of group (*P* > 0.05) indicating that levels of cocaine intake were similar between groups within females and males prior to withdrawal. Patterns of intake were also similar between the groups (group by session, *P* > 0.05) and sexes (sex by session, *P* > 0.05) with males and females in each of the groups having the highest intake during the first session [effect of session, *F*_(9,558)_ = 8.07, *P* < 0.001; session 1 versus average intake of sessions 2–10, *t*(92) = 8.56, *P* < 0.05]. Thus, while females had higher cocaine intake during the extended-access phase, there were no significant group differences in levels or patterns of cocaine intake within males or females prior to withdrawal and subsequent extinction/reinstatement testing.

#### 3.1.2. Extinction

**FIGURE 3:**
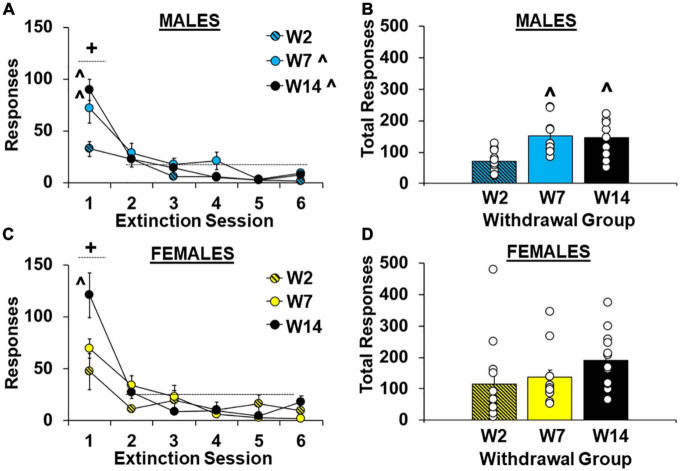
Sex- and withdrawal-specific changes in extinction responding. Mean (±SEM) number of responses on the lever formerly associated with cocaine during the first six 1-h extinction sessions **(A,C)** and across all extinction sessions completed **(B,D)** for females **(C,D)** and males **(A,B)** in the early (W2; *n* = 13 females, 10 males), intermediate (W7; *n* = 13 females, 10 males), and late (W14; *n* = 13 females, 10 males) withdrawal groups. White circles indicate individual data points. + Significantly higher responding in the first extinction session versus later ones (averaged across sessions 2–6). ^Significantly higher than the early withdrawal group (overall and/or during the first extinction session).

As predicted, cocaine-craving was significantly affected by length of withdrawal [effect of group, *F*_(2,62)_ = 4.5, *P* < 0.05] and the highest levels of extinction responding were observed in the late withdrawal group (versus the early withdrawal group, 170.2 ± 17.0 versus 95.5 ± 21.4, respectively; *P* < 0.05). Although no overall or interactive effects of sex were observed (*P*’s > 0.05, Supplementary Figure 1A), planned analyses within males and females separately indicate that the withdrawal-dependent increase in extinction responding was driven primarily by effects in males (Figures 3A–D). Specifically, within males there was a significant effect of withdrawal group for both hourly extinction responses [Figure 3A; *F*_(2,27)_ = 7.6, *P* < 0.01; η*P*^2^, 0.36] and total extinction responses across all extinction sessions run [Figure 3B; *F*_(2,27)_ = 7.6, *P* < 0.01; η*P*^2^, 0.36]. *Post hoc* comparison also confirmed higher total extinction responses in the intermediate and late withdrawal groups versus the early withdrawal group (*P*’s < 0.01). The analysis of hourly extinction responding in males also revealed a significant effect of session [*F*_(5,135)_ = 40.2, *P* < 0.001], which reflects higher responding in the first session versus later ones [session 1 versus average of sessions 2–6, *t*(29) = 7.70, *P* < 0.001], as well as an interaction of group by session [*F*_(10,135)_ = 3.4, *P* < 0.001] which reflects a group difference [*F*_(2,27)_ = 6.8, *P* < 0.01] and higher responding in the intermediate and late withdrawal groups as compared to the early withdrawal group (*P*’s < 0.05) during the first extinction session, but not later ones (*P*’s > 0.05). In contrast, within females, the effect of group was non-significant for both hourly extinction responses [Figure 3C; *F*_(2,35)_ = 1.7, *P* > 0.05] and total extinction responses across all extinction sessions run [Figure 3D; *F*_(2,35)_ = 1.8, *P* > 0.05] indicting that extinction responding was similarly increased at early versus later withdrawal time-points. However, similar to males, the analysis of hourly extinction response within females revealed a significant effect of session [*F*_(5,175)_ = 35.34, *P* < 0.001], which reflects higher responding in the first session versus later ones [sessions 2–6, *t*(37) = 9.45, *P* < 0.001], as well as an interaction of group by session [*F*_(10,175)_ = 4.63, *P* < 0.001] which reflects a group difference [*F*_(2,35)_ = 5.1, *P* < 0.05] and higher responding in the late withdrawal group compared to the early withdrawal group (*P* < 0.05) during the first extinction session. For both males and females, responding decreased to similarly low levels in each of the groups by session 6 (effect of group, *P’s* > 0.05). These findings indicate that in males, extinction responding increases over withdrawal and peaks during intermediate withdrawal (i.e., following 7 days of withdrawal). While a similar effect also occurred in females for responding during the first extinction session, total extinction responses were already elevated in females during early withdrawal (i.e., following 2 days of withdrawal) and did not further increase over withdrawal.

#### 3.1.3. Reinstatement

**FIGURE 4:**
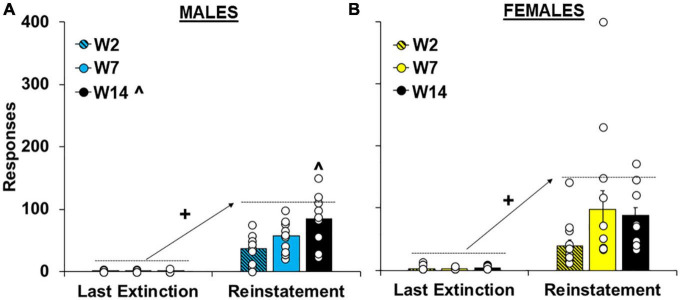
Sex- and withdrawal-specific changes in cue-induced reinstatement responding. Mean (±SEM) number of responses made on the lever formerly associated with cocaine during the last extinction session versus the reinstatement session for **(A)** males and **(B)** females the early (W2; *n* = 13 females, 10 males), intermediate (W7; *n* = 13 females, 10 males), and late (W14; *n* = 13 females, 10 males) withdrawal groups. White circles indicate individual data points. + Significant increase from the last extinction session. ^Significantly higher than the early withdrawal group.

As with extinction responding, cue-induced reinstatement of cocaine-craving was significantly affected by the length of withdrawal [effect of group, *F*_(2,62)_ = 4.6, *P* < 0.05] and the highest levels of responding were observed in the intermediate and late withdrawal groups (versus the early withdrawal group; 80.3 ± 17.6 and 86.5 ± 8.8 versus 38.0 ± 6.2, respectively; *P*’s < 0.05). Although no overall or interactive effects of sex were observed in the overall analysis (Supplementary Figure 1B), planned analyses within males and females separately indicate that, like extinction responding, withdrawal-dependent increases in reinstatement responding were driven primarily by effects in males (Figure 4A). Specifically, for males responding was significantly reinstated by the cues in each of the withdrawal groups [versus the last extinction session; effect of session, *F*_(1,27)_ = 102.4, *P* < 0.001; within the early, *t*(9) = −4.40, *P* < 0.001, intermediate, *t*(12) = −5.95, *P* < 0.001, and late, *t*(12) = −5.55, *P* < 0.001, withdrawal groups] and this reinstatement effect was significantly affected by length of withdrawal [effect of group, *F*_(2,27)_ = 6.4, *P* < 0.01; session by group interaction *F*_(2,27)_ = 5.9, *P* < 0.01]. Although no group difference was observed within the last extinction session (*P* < 0.05), there was a significant effect of group within the reinstatement session [*F*_(2,27)_ = 6.1, *P* < 0.01; η*P*^2^, 0.13] with subsequent *post hoc* comparisons revealing a significant difference between the early and the late (*P* < 0.01) withdrawal groups. In contrast, the same analysis within females revealed a significant effect of session [Figure 4B; *F*_(1,35)_ = 40.5, *P* < 0.001], but non-significant trends for group [*F*_(1,35)_ = 2.47, *P* = 0.10] and session by group [*F*_(1,35)_ = 2.63, *P* = 0.09]. *Post hoc* analyses also confirmed non-significant group differences within both the last extinction session (*P* > 0.05) and the reinstatement session (*P* > 0.05). Together, these findings indicate that in males, cue-induced cocaine-craving progressively increases from early to late withdrawal (following 2 versus 14 days) whereas in females, cocaine-craving is already elevated during early withdrawal (following 2 days) and does not further increase over withdrawal.

Given the sex difference in cocaine intake during the extended-access period, we ran correlational analyses to determine whether cocaine intake was predictive of later cocaine-craving during extinction and reinstatement testing (total responses for each; data not shown). These associations were non-significant for both extinction (*r* = 0.04) and cue-induced reinstatement (*r* = 0.13) in the overall sample and within females (*r* = −0.08 and 0.11, respectively) and males (*r* = 0.10 and 0.04, respectively) separately. These findings indicate that prior cocaine intake was not predictive of later cocaine-craving.

### 3.2. Molecular results

#### 3.2.1. *Bdnf-IV* gene expression

**FIGURE 5:**
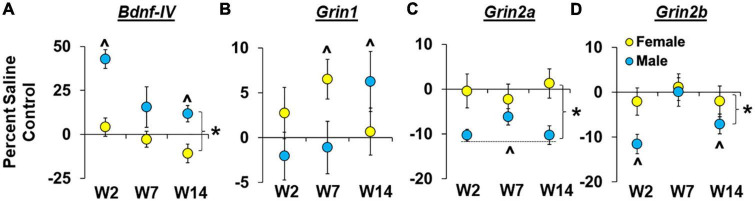
Sex- and withdrawal-specific changes in *Bdnf-IV*, *Grin1*, *Grin2a*, and *Grin2b* expression in the dorsomedial prefrontal cortex (dmPFC). Mean (±SEM) percent difference from saline controls for *Bdnf-IV* **(A)**, *Grin1* **(B)**, *Grin2a* **(C)**, *Grin2b* **(D)** in the dmPFC for males and females in the early (W2; *n* = 13 females, 10 males), intermediate (W7; *n* = 13 females, 10 males), and late (W14; *n* = 13 females, 10 males) withdrawal groups. *Significant difference between females and males. ^Significant difference from zero in females and males.

Sex- and withdrawal-dependent effects were observed for *Bdnf-IV* expression within the dmPFC (Figure 5A) with results from the analysis of percent difference from saline controls, which normalizes baseline sex differences, revealing that males had a larger increase than females in *Bndf-IV* expression [effect of sex, *F*_(1,60)_ = 27.7, *P* < 0.001; η*P*^2^, 0.30]. Effects were most pronounced in males in the early withdrawal group [effect of group, *F*_(2,60)_ = 6.9, *P* < 0.01; early withdrawal group versus 0, *P* < 0.05], and while the interaction of sex and withdrawal group was not statistically significant (*P* > 0.05), results from the planned analysis within each sex confirmed an effect of withdrawal group within males [*F*_(2,4.4)_ = 4.4, *P* < 0.05]. Within males, the increase in *Bdnf-IV* expression was also significantly greater than zero for both the early and late withdrawal groups (*P* < 0.05’s). In contrast, the effect of withdrawal group was not significant in females (*P* > 0.05), and collapsed across withdrawal groups, expression levels were not significantly different from zero (*P* > 0.05). Thus, *Bdnf-IV* expression was increased following relapse testing in males, but not females, and while effects were most pronounced following testing during early withdrawal, expression remained elevated persistently (i.e., following relapse testing and 14 days of withdrawal).

#### 3.2.2. *Grin1* gene expression

Sex- and withdrawal-dependent effects were observed for *Grin1* expression within the dmPFC (Figure 5B) with results from the analysis of percent difference from saline controls revealing non-significant overall effects of sex and withdrawal group but a trend for a significant interaction of sex by withdrawal group [Figure 5D; *F*_(2,62)_ = 3.1, *P* = 0.053; η*P*^2^, 0.09]. Further analysis within each sex revealed non-significant effects of withdrawal group for both males and females (*P*’s > 0.05) although within females there was a trend for increased *Grin1* expression (>0, relative to saline controls, *P* = 0.06) which appears to be driven by effects within the intermediate withdrawal group given that expression was significantly higher than saline controls in the intermediate withdrawal group (*P* < 0.05), but not in the early or late groups. The same analysis within males showed that *Grin1* expression was also increased relative to saline controls, but in contrast to effects in females, this occurred in the late withdrawal group in males (*P* < 0.05), not the intermediate group (or early group). Thus, females appear to have higher *Grin1* expression following relapse testing during intermediate withdrawal, while, males showed an increase in *Grin1* expression during late withdrawal.

#### 3.2.3. *Grin2a* gene expression

Sex-dependent effects were observed for *Grin2a* expression within the dmPFC (Figure 5C) with results from the analysis of percent difference from saline controls revealing a significant overall effect of sex [*F*_(1,59)_ = 10.8, *P* < 0.01; η*P*^2^, 0.15] indicating that males show a greater decrease in *Grin2a* expression than females following relapse testing (*P* < 0.01). Further analysis in males revealed a non-significant effect of withdrawal group (*P* > 0.05) and a significant difference from zero for percent of control expression when collapsed across withdrawal groups (*P* < 0.001), indicating that the decrease in *Grin2a* expression was consistent across each of the withdrawal time-points. In females, the overall effect of withdrawal group was also non-significant (*P* > 0.05), but in contrast to males, in females, percent of control *Grin2a* expression was not significantly different from zero (*P* > 0.05). Thus, *Grin2a* expression was decreased following relapse testing during early, intermediate, and late withdrawal in males but was unchanged at these withdrawal time-points in females.

#### 3.2.4. *Grin2b* gene expression

Sex- and withdrawal-dependent effects were observed for *Grin2b* expression within the dmPFC (Figure 5D) with results from the analysis of percent difference from saline controls revealing a significant effect of sex [*F*_(1,60)_ = 4.4, *P* < 0.05; η*P*^2^, 0.07] indicating that males had a greater decrease in *Grin2b* expression (relative to saline controls) than females following relapse testing. There was also a significant effect of withdrawal group [*F*_(2,60)_ = 3.2, *P* < 0.05] that again appears to be driven by changes in males given that follow-up analysis within each sex revealed a significant effect of withdrawal group for males [*F*_(2,25)_ = 5.2, *P* < 0.05], but not females (*P* > 0.05). Further analysis within males also showed that the decrease in *Grin2b* expression (relative to saline controls) was significantly lower than zero for both the early (*P* < 0.01) and late (*P* < 0.05) withdrawal groups. Within females, the change in percent of control *Grin2b* expression was not significantly different from zero (*P* > 0.05). Thus, *Grin2b* expression in the dmPFC decreased following relapse testing in males, but not females, and while effects were most pronounced following testing during early withdrawal, modest decreases were also observed during late withdrawal.

## 4. Discussion

Women have a shorter duration of cocaine use prior to developing problematic cocaine use and show an enhanced vulnerability to cocaine-related medical consequences including a shorter time interval between onset of cocaine use and a fatal outcome compared to men (White et al., 1996; Sofuoglu et al., 1999; Haas and Peters, 2000; O’Brien and Anthony, 2005; Origer et al., 2014; see Agabio et al., 2016 for review). These sex difference appear to be driven by biological differences considering our previous findings showing that a parallel phenomenon also occurs in female rats with the development of two key features of cocaine use disorder, an enhanced motivation for cocaine and compulsive cocaine use despite negative consequences (Towers et al., 2021). Here, we expanded on the investigation of the telescoping effect by determining whether the time-course for the incubation of cocaine-craving, another key feature of cocaine use disorder in humans, also develops sooner during withdrawal in females than males. We found that in males, cocaine-craving (total extinction and cue-induced reinstatement responding) progressively increased from early to later periods of withdrawal whereas in females, cocaine-craving was already elevated during early withdrawal (following 2 days of withdrawal) and did not further increase over withdrawal (i.e., non-significant effect of withdrawal group). While these findings are consistent with our hypothesis for a faster course for the incubation of cocaine-craving in females, this effect was modest considering it was only apparent in the sex-specific time-course analyses (i.e., there were no overall or interactive effects of sex on levels of cocaine-craving). Cocaine-craving during the first hour of extinction testing also similarly increased from early to later periods of withdrawal in both males and females. Despite these modest sex differences in behavior, we observed marked sex differences in the molecular adaptations associated with the incubation of cocaine-craving. Specifically, we showed that *Bdnf-IV*, *Grin2a*, *Grin2b*, and *Grin1* gene expression changed in response to withdrawal and relapse testing in males, while females only showed a modest increase in the expression of *Grin1* at the intermediate withdrawal time-point. Together, these findings provide evidence for a modestly faster time-course for the development of incubated cocaine-craving in females versus males, and indicate that the underlying molecular adaptations differ in females versus males. Each of these molecular findings are discussed further below.

A large body of work conducted in males has shown that cue-induced drug-craving increases, or incubates, from early (days 1–2) to later periods of withdrawal (days 14–60; Grimm et al., 2001; Li et al., 2015). A similar effect has been confirmed to occur in females with findings showing that males and females have similarly low levels of cue-induced cocaine-craving during early withdrawal (on withdrawal day 1); high levels of cue-induced cocaine-craving are reported in males and females during later withdrawal time-points (following 14 and 48 days of withdrawal), with the highest levels observed in females tested during estrus (versus males and females tested during non-estrus phases; Nicolas et al., 2019; Corbett et al., 2021). Our current results build on this body of literature and show that there is also a modest sex difference on the ascending limb of the incubation of cocaine-craving curve, with elevated cue-induced cocaine-craving already evident in females during early withdrawal (following 2 days of withdrawal). This conclusion is also consistent with findings from Nicolas et al. (2019) showing that in females, levels of cue-induced cocaine-craving during late withdrawal (day 29) was not significantly higher than those observed during early withdrawal (day 2) indicating that levels of cocaine-craving were already elevated in females during early withdrawal. However, as with our effects here, the effect in this previous study was modest and observed in only one of the two cohorts tested suggesting that the 2-day withdrawal time-point is a threshold condition for females to start expressing high levels of cocaine-craving. This idea is also consistent with findings showing that cocaine-craving is low in both males and females immediately following extended-access self-administration (day 1 of withdrawal), but then increases in both sexes by withdrawal day 15 (Corbett et al., 2021). It is also consistent with our findings showing that motivation for cocaine, as assessed under a progressive-ratio schedule, is either unchanged or decreased from baseline in females and males tested immediately following extended-access self-administration (day 1 of withdrawal), but then increases in both males and females tested following 14 or more days of withdrawal (Lynch and Taylor, 2005; Towers et al., 2021). Together, these findings suggest that cocaine-craving incubates over withdrawal in both sexes, but that in females, incubation occurs sooner such that by withdrawal day 3, cocaine-craving is already increased from low levels. Future studies are necessary to confirm this possibility.

Our findings on incubation of cocaine-craving are also consistent with previous results from our group and others showing that females develop other features of an addiction-like phenotype, including an enhanced motivation for cocaine, compulsive cocaine use despite punishment, and cocaine preference over other non-drug rewards, sooner during withdrawal or after less drug exposure than males (Lynch and Taylor, 2004; Kerstetter et al., 2012; Perry et al., 2013, 2015; Kawa and Robinson, 2019; Towers et al., 2021). This effect with cocaine-craving does appear to be less robust as compared to effects reported for the development of other addiction-like features. For example, with the development of an enhanced motivation for the cocaine, females, but not males, develop this phenotype under threshold conditions (following extended-access self-administration and 7 days of withdrawal), with females responding at roughly 25% higher levels than males to obtain infusions of cocaine. In contrast, the effect here with cocaine-craving was observed for total extinction and cue-induced reinstatement responding, but not for the first hour of extinction responding which increased progressively in both sexes from early to later periods of withdrawal.

These sex differences in the incubation of cocaine-craving do not appear to be driven by females having great cocaine intake during the extended-access period considering that cocaine intake was not predictive of subsequent cocaine-craving, which is consistent with our previous findings for the development of enhanced motivation for cocaine and compulsive cocaine use despite negative consequences (Towers et al., 2021). This idea is also supported by findings from the two previous studies on sex differences in cue-induced cocaine-craving (Nicolas et al., 2019; Corbett et al., 2021) which showed that females had higher levels of cue-induced craving during late withdrawal despite self-administering similar levels of cocaine during the extended-access phase. It is notable that intake did not differ between the sexes in the previous studies given that these studies also used extended-access conditions [i.e., 6-h/day with the long-access procedure, Corbett et al. (2021); 8-h/day using a different intermittent-access procedure, Nicolas et al., 2019]. Both studies also used low to moderate doses of cocaine (i.e., 0.5 or 0.75 mg/kg/infusion) which are typically more sensitive to individual differences, such as sex, than high doses like the one used here (1.5 mg/kg). None-the-less, we have consistently observed higher intake in females than males under these extended-access conditions (e.g., Doyle et al., 2014; Peterson A. et al., 2014; Towers et al., 2021) suggesting that our procedure is sensitive to sex differences in intake.

One limitation to our findings is that we were not able to consider the impact of the estrous cycle on the time-course for the incubation of cocaine-craving in females. Estrous cycle phase is known to impact drug-craving during later (day 15 and 48), but not early (day 1) withdrawal time-points with estrus females having higher levels of cocaine-craving than males, but no difference between non-estrus females and males (Nicolas et al., 2019; Corbett et al., 2021). Considering that overall levels of craving were similar between males and females in the current study, it is likely that the majority of our females were tested during a non-estrus phase of their cycle. However, future studies are necessary to determine whether the phase of the estrous cycle impacts cocaine-craving following 2 days of withdrawal and whether ovarian hormones contribute to the modestly accelerated time-course for the incubation of cocaine craving in females. This question is important considering evidence indicating that estradiol underlies the accelerated course in females for the development of other key addiction-like features, such as an enhanced motivation for cocaine (Ramôa et al., 2013, 2014; Bakhti-Suroosh et al., 2019).

Our molecular findings are suggestive of an earlier recruitment of glutamatergic signaling in the dmPFC of females than males, which is critical for the incubation of cocaine-craving in both humans and animal models (Rebec and Sun, 2005; Goldstein and Volkow, 2011; Kalivas and Volkow, 2011; Szumlinski and Shin, 2018). Specially, *Grin1*, the gene that encodes the ubiquitous subunit of NMDA receptor (i.e., GluN1), was increased sooner in withdrawal in females than males (during intermediate versus late withdrawal). While this effect was modest and only apparent in the within-sex analyses, it does mirror our behavioral findings of an accelerated course to incubated craving in females; however, the molecular shift is delayed compared to the behavior, which is likely the result of the early withdrawal timepoint being threshold for females to start expressing high levels of cocaine-craving. This idea is further supported by findings in males where total extinction response reaches peak-levels following 7 days of withdrawal, but the molecular shift in *Grin1* does not occur until after 14 days of withdrawal. Our findings are also consistent with previous findings with methamphetamine showing that a molecular shift toward increased NMDA receptor currents in the PFC occurs after less extended-access self-administration in females than males (Mishra et al., 2017; Pena-Bravo et al., 2019). These previous studies further showed that the increase in NMDA receptor currents that developed following withdrawal from extended-access methamphetamine self-administration was likely due to changes in GluN2B signaling in the PFC males, whereas, it was not affected by GluN2B antagonism in the PFC in females (Mishra et al., 2017; Pena-Bravo et al., 2019). These findings are similar to our observations here that *Grin2b*, the gene that encodes GluN2B subunit of the NMDA receptor, was decreased in males during both early and late withdrawal, but unchanged across withdrawal in females. These findings are intriguing because they suggest that some of the molecular adaptations associated with development of substance use disorder may be accelerated (*Grin1*), while others are qualitatively different in females compared to males (*Grin2a* and *Grin2b*).

Although *Bdnf-IV*, *Grin2a*, and *Grin2b* expression did not change in response to withdrawal and relapse testing in females, our molecular results in males are consistent with previous findings indicating that in males, BDNF and NMDA receptor subunits play a critical role in the incubation of cocaine-craving. More specifically, for *Bdnf-IV*, we observed increase expression in the dmPFC of males following relapse testing during early and late withdrawal. These findings are consistent with our previous findings from two separate studies showing that *Bdnf-IV* expression in the dmPFC is elevated in males following withdrawal from extended-access cocaine self-administration and cue-induced relapse testing (day 15; Peterson A. et al., 2014; Abel et al., 2019). We also previously showed that exercise (i.e., wheel running) during withdrawal dose dependently attenuated both relapse responding and *Bdnf-IV* expression (Peterson A. et al., 2014), which provides further support for dmPFC BDNF-IV contributing to the incubation of cocaine-craving/relapse vulnerability in males. It is notable that the greatest increase in *Bdnf-IV* expression observed in males in the current study was following relapse testing during early withdrawal, when levels of cocaine-craving were low. This is surprising considering that *Bdnf-IV* expression presumably increases progressively over withdrawal. Our current findings suggest that this is not the case; this idea is also consistent with clinical findings in individuals with cocaine use disorder showing that high levels of serum BDNF during early withdrawal were predictive of early relapse following discharge from an inpatient abstinence program (within ∼4 days; Corominas-Roso et al., 2015). Individuals with high levels of BDNF during early withdrawal also did not show withdrawal-dependent increases in serum BDNF (Corominas-Roso et al., 2015) which is in contrast to individuals with lower levels of serum BDNF during early withdrawal who did show an increase serum levels of BNDF over withdrawal (Corominas-Roso et al., 2015). Together, these findings suggest that increased *Bdnf-IV* expression in the dmPFC can precede the development of incubated cocaine-craving; BDNF is also likely a clinically relevant marker for early relapse. However, considering that the clinical data were obtained from a predominantly male sample (only 2 of the 40 patients were women; Corominas-Roso et al., 2015), along with our current findings showing that *Bdnf-IV* expression did not change in response to withdrawal and relapse testing in females, it is possible that findings with BDNF apply to males, but not females. Future research is necessary to address this possibility.

Similarly, our findings in males for NMDA receptor subunit expression are consistent with a large body of work indicating that NMDA receptors are causally involved in the incubation of cocaine-craving in males (Szumlinski and Shin, 2018). Here, we confirmed that expression levels of *Grin1*, *Grin2a*, and *Grin2b* are low following cue-induced relapse testing during early withdrawal (after 2 days), when cocaine-craving was the lowest in males (also see Ben-Shahar et al., 2009; Szumlinski et al., 2016; Barry and McGinty, 2017), and that expression of *Grin1* was increased following cue-induced relapse testing during late withdrawal (after 14 days), when cocaine-craving was the highest in males (also see Abel et al., 2019). These findings are also consistent with the pathophysiology of substance use disorder in humans, which is associated with increased levels of GluN1 expression, the protein encoded by *Grin1* (Enoch et al., 2014; Daneshparvar et al., 2019). However, it is surprising that *Grin2b* expression levels continued to be decreased during late withdrawal considering findings showing that GluN2B protein levels in the dmPFC become increased in response to drug-associated cues as early as day 3 of withdrawal and remain increased up to 30 days of withdrawal (Szumlinski et al., 2016). The reason for the discrepancy between *Grin2b* mRNA expression and the protein it encodes, GluN2B, is unknown but may be the result of procedural differences, or more likely, a difference in gene versus protein expression. GluN2A protein levels also increase from low levels within the first week of withdrawal from extended-access self-administration, but do not become elevated until 60 days of withdrawal (Ben-Shahar et al., 2009; Szumlinski et al., 2016). A limitation to this study is that we only investigated differences at the mRNA level; thus, future studies will need to determine differences at the protein level.

In conclusion, our findings indicate that the incubation of cocaine-craving develops more rapidly in females than males, although this effect is modest and overall levels of cocaine-craving were similar between the sexes. Despite the modest sex difference in behavior, there were marked differences between males and females in the molecular adaptations known to mediate cocaine-craving in males, and some of these differences (i.e., *Grin1*) parallel the behavioral differences. These findings highlight a need for further research on sex differences in mechanism underlying cocaine use disorder as this information is needed to develop sex-specific prevention and treatment strategies.

## Data availability statement

The raw data supporting the conclusions of this article will be made available by the authors, without undue reservation.

## Ethics statement

The animal study was reviewed and approved by the University of Virginia Animal Care and Use Committee.

## Author contributions

ET and WL designed the study and wrote the manuscript. ET, WL, MK, and IW performed the statistical analysis. AB-S, LP, and JA collected the data. All authors contributed to the manuscript revision, read, and approved the submitted version.

## Supplementary material

Supplementary Figure 1 Withdrawal-specific changes in cocaine craving. Mean (±SEM) number of responses on the lever formerly associated with cocaine across all extinction sessions completed **(A)** and during the last extinction session versus the reinstatement session **(B)** in females and males in the early (W2; *n* = 13 females, 10 males), intermediate (W7; *n* = 13 females, 10 males), and late (W14; *n* = 13 females, 10 males) withdrawal groups. ^ Significant difference from early withdrawal group.

## References

[B1] Abel J. Nesil T. Bakhti-Suroosh A. Grant P. Lynch W. (2019). Mechanisms underlying the efficacy of exercise as an intervention for cocaine relapse: A focus on mGlu5 in the dorsal medial prefrontal cortex. *Psychopharmacology* 236 2155–2171. 10.1007/s00213-019-05208-0 31161451 PMC6626681

[B2] Adelson M. Linzy S. Peles E. (2018). Characteristics and outcome of male and female methadone maintenance patients: MMT in Tel Aviv and Las Vegas. *Subst. Use Misuse* 53 230–238. 10.1080/10826084.2017.1298619 28574738

[B3] Agabio R. Campesi I. Pisanu C. Gessa G. Franconi F. (2016). Sex differences in substance use disorders: Focus on side effects. *Addict. Biol.* 21 1030–1042. 10.1111/adb.12395 27001402

[B4] Algallal H. Allain F. Ndiaye N. Samaha A. (2020). Sex differences in cocaine self-administration behaviour under long access versus intermittent access conditions. *Addict. Biol.* 25:e12809. 10.1111/adb.12809 31373148

[B5] Anglin M. Hser Y. McGlothlin W. (1987). Sex differences in addict careers. 2. Becoming addicted. *Am. J. Drug Alcohol. Abuse* 13 59–71. 10.3109/00952998709001500 3687885

[B6] Back S. Payne R. Wahlquist A. Carter R. Stroud Z. Haynes L. (2011a). Comparative profiles of men and women with opioid dependence: Results from a national multisite effectiveness trial. *Am. J. Drug Alcohol. Abuse* 37 313–323. 10.3109/00952990.2011.596982 21854273 PMC3164783

[B7] Back S. Lawson K. Singleton L. Brady K. (2011b). Characteristics and correlates of men and women with prescription opioid dependence. *Addict. Behav.* 36 829–834. 10.1016/j.addbeh.2011.03.013 21514061 PMC3164361

[B8] Bakhti-Suroosh A. Nesil T. Lynch W. (2019). Tamoxifen blocks the development of motivational features of an addiction-like phenotype in female rats. *Front. Behav. Neurosci.* 13:253. 10.3389/fnbeh.2019.00253 31780909 PMC6856674

[B9] Barry S. McGinty J. (2017). Role of Src family kinases in BDNF-mediated suppression of cocaine-seeking and prevention of cocaine-induced ERK, GluN2A, and GluN2B dephosphorylation in the prelimbic cortex. *Neuropsychopharmacology* 42 1972–1980. 10.1038/npp.2017.114 28585567 PMC5561338

[B10] Becker J. (2009). Sexual differentiation of motivation: A novel mechanism? *Horm Behav.* 55 646–654. 10.1016/j.yhbeh.2009.03.014 19446081 PMC2684520

[B11] Becker J. B. Koob G. F. (2016). Sex differences in animal models: Focus on addiction. *Pharmacol. Rev.* 68 242–263. 10.1124/pr.115.011163 26772794 PMC4813426

[B12] Becker J. McClellan M. Reed B. (2016). Sociocultural context for sex differences in addiction. *Addict. Biol.* 21 1052–1059. 10.1111/adb.12383 26935336 PMC5555215

[B13] Beiter R. Peterson A. Abel J. Lynch W. (2016). Exercise during early, but not late abstinence, attenuates subsequent relapse vulnerability in a rat model. *Transl. Psychiatry* 6:e792. 10.1038/tp.2016.58 27115123 PMC4872415

[B14] Beltz A. Beery A. Becker J. (2019). Analysis of sex differences in pre-clinical and clinical data sets. *Neuropsychopharmacology* 44 2155–2158. 10.1038/s41386-019-0524-3 31527863 PMC6898365

[B15] Ben-Shahar O. Obara I. Ary A. Ma N. Mangiardi M. Medina R. (2009). Extended daily access to cocaine results in distinct alterations in Homer 1b/c and NMDA receptor subunit expression within the medial prefrontal cortex. *Synapse* 63 598–609. 10.1002/syn.20640 19306440 PMC2749486

[B16] Ben-Shahar O. Sacramento A. Miller B. Webb S. Wroten M. Silva H. (2013). Deficits in ventromedial pre- frontal cortex group1 metabotropic glutamate receptor function mediate resistance to extinction during protracted withdrawal from an extensive history of cocaine self-administration. *J. Neurosci.* 33 495a–506a. 10.1523/JNEUROSCI.3710-12.2013 23303930 PMC3711633

[B17] Ben-Shahar O. Szumlinski K. Lominac K. Cohen A. Gordon E. Ploense K. (2012). Extended access to cocaine self-administration results in reduced glutamate function within the medial prefrontal cortex. *Addict. Biol.* 17 746–757. 10.1111/j.1369-1600.2011.00428.x 22339852 PMC3360114

[B18] Brecht M. O’Brien A. von Mayrhauser C. Anglin M. (2004). Methamphetamine use behaviors and gender differences. *Addict. Behav.* 29 89–106. 10.1016/S0306-4603(03)00082-0 14667423

[B19] Caffino L. Verheij M. Roversi K. Targa G. Mottarlini F. Popik P. (2020). Hypersensitivity to amphetamine’s psychomotor and reinforcing effects in serotonin transporter knockout rats: Glutamate in the nucleus accumbens. *BJP* 177 4532–4547. 10.1111/bph.15211 32721055 PMC7484509

[B20] CDC WONDER (2022). *Centers for disease control and prevention.* Available online at: https://wonder.cdc.gov/ (accessed July 1, 2022).

[B21] Center for Substance Abuse Treatment (2009). *Substance abuse treatment: Addressing the specific needs of women SAMHSA/CSAT treatment improvement protocols.* Rockville, MD: Substance Abuse and Mental Health Services Administration), 1–48. 22514859

[B22] Chen Y. Barson J. Chen A. Hoebel B. Leibowitz S. (2013). Glutamatergic input to the lateral hypothalamus stimulates ethanol intake: Role of orexin and melanin-concentrating hormone. *Alcoholism* 37 123–131. 10.1111/j.1530-0277.2012.01854.x 22823322 PMC4007270

[B23] Corbett C. Dunn E. Loweth J. (2021). Effects of sex and estrous cycle on the time-course of incubation of cue-induced craving following extended-access cocaine self-administration. *eNeuro* 8. 10.1523/ENEURO.0054-21.2021 34290059 PMC8362687

[B24] Corominas-Roso M. Roncero C. Daigre C. Grau-Lopez L. Ros-Cucurull E. Rodríguez-Cintas L. (2015). Changes in brain-derived neurotrophic factor (BDNF) during abstinence could be associated with relapse in cocaine-dependent patients. *Psychiatry Res.* 225 309–314. 10.1016/j.psychres.2014.12.019 25592977

[B25] Corominas-Roso M. Roncero C. Eiroa-Orosa F. Gonzalvo B. Grau-Lopez L. Ribases M. (2013). Brain-derived neurotrophic factor serum levels in cocaine-dependent patients during early abstinence. *Eur. Neuropsychopharmacol.* 23 1078–1084. 10.1016/j.euroneuro.2012.08.016 23021567

[B26] Cyr M. Ghribi O. Thibault C. Morissette M. Landry M. Di Paolo T. (2001). Ovarian steroids and selective estrogen receptor modulators activity on rat brain NMDA and AMPA receptors. *Brain Res. Brain Res. Rev.* 37 153–161. 10.1016/S0165-0173(01)00115-1 11744083

[B27] Daneshparvar H. Sadat-Shirazi M. S. Fekri M. Khalifeh S. Ziaie A. Esfahanizadeh N. (2019). NMDA receptor subunits change in the prefrontal cortex of pure-opioid and multi-drug abusers: A post-mortem study. *Eur. Arch. Psychiatry Clin. Neurosci.* 269 309–315. 10.1007/s00406-018-0900-8 29766293

[B28] DiFranza J. Savageau J. Rigotti N. Fletcher K. Ockene J. McNeill A. (2002). Development of symptoms of tobacco dependence in youths: 30 month follow up data from the DANDY study. *Tob. Control* 11 228–235. 10.1136/tc.11.3.228 12198274 PMC1759001

[B29] Doyle S. Ramôa C. Garber G. Newman J. Toor Z. Lynch W. (2014). A shift in the role of glutamatergic signaling in the nucleus accumbens core with the development of an addicted phenotype. *Biol. Psychiatry* 76 810–815. 10.1016/j.biopsych.2014.02.005 24629536 PMC4133320

[B30] D’Sa C. Fox H. Hong A. Dileone R. Sinha R. (2011). Increased serum brain-derived neurotrophic factor is predictive of cocaine relapse outcomes: A prospective study. *Biol. Psychiatry* 70 706–711. 10.1016/j.biopsych.2011.05.013 21741029 PMC3186871

[B31] Ehlers C. Gizer I. Vieten C. Gilder D. Stouffer G. Lau P. (2010). Cannabis dependence in the San Francisco family study: Age of onset of use. DSM-IV symptoms, withdrawal, and heritability. *Addict. Behav.* 35 102–110. 10.1016/j.addbeh.2009.09.009 19818563 PMC2805269

[B32] Enoch M. A. Rosser A. A. Zhou Z. Mash D. C. Yuan Q. Goldman D. (2014). Expression of glutamatergic genes in healthy humans across 16 brain regions; altered expression in the hippocampus after chronic exposure to alcohol or cocaine. *Genes Brain Behav.* 13 758–768. 10.1111/gbb.12179 25262781 PMC4241133

[B33] Funk D. Coen K. Tamadon S. Hope B. Shaham Y. Le A. (2016). Role of central amygdala neuronal ensembles in incubation of nicotine craving. *J. Neurosci.* 36 8612–8623. 10.1523/JNEUROSCI.1505-16.2016 27535909 PMC4987435

[B34] Gallop R. Crits-Christoph P. Ten Have T. Barber J. Frank A. Griffin M. (2007). Differential transitions between cocaine use and abstinence for men and women. *J. Consult. Clin. Psychol.* 75 95–103. 10.1037/0022-006X.75.1.95 17295568

[B35] Gancarz-Kausch A. Adank D. Dietz D. (2014). Prolonged withdrawal following cocaine self-administration increases resistance to punishment in a cocaine binge. *Sci. Rep.* 4:6876. 10.1038/srep06876 25363133 PMC4217113

[B36] Goldstein R. Volkow N. (2011). Dysfunction of the prefrontal cortex in addiction: Neuroimaging findings and clinical implications. *Nat. Rev. Neurosci.* 12 652–669. 10.1038/nrn3119 22011681 PMC3462342

[B37] Grant J. Odlaug B. Mooney M. (2012). Telescoping phenomenon in pathological gambling: Association with gender and comorbidities. *J. Nerv. Ment. Dis.* 200 996–998. 10.1097/NMD.0b013e3182718a4d 23124186 PMC3499774

[B38] Greenfield S. Back S. Lawson K. Brady K. (2010). Substance abuse in women. *Psychiatr. Clin. North Am* 33 339–355. 10.1016/j.psc.2010.01.004 20385341 PMC3124962

[B39] Griffin M. Weiss R. Mirin S. Lange U. (1989). A comparison of male and female cocaine abusers. *Arch. Gen. Psychiatr.* 46 122–126. 10.1001/archpsyc.1989.01810020024005 2913971

[B40] Grimm J. Hope B. Wise R. Shaham Y. (2001). Neuroadaptation. Incubation of cocaine craving after withdrawal. *Nature* 412 141–142. 10.1038/35084134 11449260 PMC2889613

[B41] Haas A. Peters R. (2000). Development of substance abuse problems among drug-involved offenders: Evidence for the telescoping effect. *J. Subst. Abuse* 12 241–253. 10.1016/S0899-3289(00)00053-5 11367602

[B42] Hearing M. Graziane N. Dong Y. Thomas M. (2018). Opioid and psychostimulant plasticity: Targeting overlap in nucleus accumbens glutamate signaling. *Trends Pharmacol. Sci.* 39 276–294. 10.1016/j.tips.2017.12.004 29338873 PMC5818297

[B43] Hernandez-Avila C. Rounsaville B. Kranzler H. (2004). Opioid-, cannabis- and alcohol-dependent women show more rapid progression to substance abuse treatment. *Drug Alcohol Depend.* 74 265–272. 10.1016/j.drugalcdep.2004.02.001 15194204

[B44] Hser Y. Anglin M. McGlothlin W. (1987a). Sex differences in addict careers. 3. Addiction. *Am. J. Drug Alcohol. Abuse* 13 231–251. 10.3109/00952998709001512 3687889

[B45] Hser Y. Anglin M. McGlothlin W. (1987b). Sex differences in addict careers. 1. Initiation of use. *Am. J. Drug Alcohol Abuse* 13 33–57. 10.3109/00952998709001499 3318399

[B46] Ibanez A. Blanco C. Moreryra P. Saiz-Ruiz J. (2003). Gender differences in pathological gambling. *J. Clin. Psychiatry* 64 295–301. 10.4088/JCP.v64n0311 12716271

[B47] Ishikawa A. Ambroggi F. Nicola S. Fields H. (2008). Dorsomedial prefrontal cortex contribution to behavioral and nucleus accumbens neuronal responses to incentive cues. *J. Neurosci.* 28 5088–5098. 10.1523/JNEUROSCI.0253-08.2008 18463262 PMC2661106

[B48] Jackson L. Robinson T. Becker J. (2006). Sex differences and hormonal influences on acquisition of cocaine self-administration in rats. *Neuropsychopharmacology* 31 129–138. 10.1038/sj.npp.1300778 15920500

[B49] Kalivas P. McFarland K. (2003). Brain circuitry and the reinstatement of cocaine-seeking behavior. *Psychopharmacology* 168 44–56. 10.1007/s00213-003-1393-2 12652346

[B50] Kalivas P. Volkow N. (2011). New medications for drug addiction hiding in glutamatergic neuroplasticity. *Mol. Psychiatry* 16 974–986. 10.1038/mp.2011.46 21519339 PMC3192324

[B51] Kawa A. Robinson T. (2019). Sex differences in incentive-sensitization produced by intermittent access cocaine self-administration. *Psychopharmacology* 236 625–639. 10.1007/s00213-018-5091-5 30368583 PMC6401254

[B52] Kerstetter K. Ballis M. Duffin-Lutgen S. Carr A. Behrens A. Kippin T. (2012). Sex differences in selecting between food and cocaine reinforcement are mediated by estrogen. *Neuropsychopharmacology* 37 2605–2614. 10.1038/npp.2012.99 22871910 PMC3473343

[B53] Khan S. Secades-Villa R. Okuda M. Wang S. Perez-Fuentes G. Kerridge B. (2013). Gender differences in cannabis use disorders: Results from the national epidemic survey of alcohol and related conditions. *Drug Alcohol Depend.* 130 101–108. 10.1016/j.drugalcdep.2012.10.015 23182839 PMC3586748

[B54] Koob G. Volkow N. (2016). Neurobiology of addiction: A neurocircuitry analysis. *Lancet Psychiatry* 3 760–773. 10.1016/S2215-0366(16)00104-8 27475769 PMC6135092

[B55] Ladd G. Petry N. (2002). Gender differences among pathological gamblers seeking treatment. *Exp. Clin. Psychopharmacol.* 10 302–309. 10.1037/1064-1297.10.3.302 12233991

[B56] Le Saux M. Estrada-Camarena E. Di Paolo T. (2006). Selective estrogen receptor-alpha but not -beta agonist treatment modulates brain alpha-amino-3-hydroxy-5-methyl-4-isoxazolepropionic acid receptors. *J. Neurosci. Res.* 84 1076–1084. 10.1002/jnr.21007 16937413

[B57] Lewis B. Hoffman L. Nixon S. (2014). Sex differences in drug use among polysubstance users. *Drug Alcohol Depend.* 145 127–133. 10.1016/j.drugalcdep.2014.10.003 25454410 PMC4254592

[B58] Li X. Caprioli D. Marchant N. (2015). Recent updates on incubation of drug craving: A mini-review. *Addict. Biol.* 20 872–876. 10.1111/adb.12205 25440081 PMC4451451

[B59] Luchansky B. He L. J. Krupski A. Stark K. D. (2000). Predicting readmission to substance abuse treatment using state information systems. The impact of client and treatment characteristics. *J. Subst. Abuse* 12 255–270. 10.1016/S0899-3289(00)00055-9 11367603

[B60] Lynch W. (2008). Acquisition and maintenance of cocaine self-administration in adolescent rats: Effects of sex and gonadal hormones. *Psychopharmacology* 197 237–246. 10.1007/s00213-007-1028-0 18066534

[B61] Lynch W. Taylor J. (2004). Sex differences in the behavioral effects of 24-h/day access to cocaine under a discrete trial procedure. *Neuropsychopharmacology* 29 943–951. 10.1038/sj.npp.1300389 14872204

[B62] Lynch W. Taylor J. (2005). Decreased motivation following cocaine self-administration under extended access conditions: Effects of sex and ovarian hormones. *Neuropsychopharmacology* 30 927–935. 10.1038/sj.npp.1300656 15647749

[B63] Lynch W. Piehl K. Acosta G. Peterson A. Hemby S. (2010). Aerobic exercise attenuates reinstatement of cocaine-seeking behavior and associated neuroadaptations in the prefrontal cortex. *Biol. Psychiatry* 68 774–777. 10.1016/j.biopsych.2010.06.022 20692647 PMC2949528

[B64] Lynch W. Tan L. Narmeen S. Beiter R. Brunzell D. (2019). Exercise or saccharin during abstinence block estrus-induced increases in nicotine-seeking. *Physiol. Behav.* 203 33–41. 10.1016/j.physbeh.2017.10.026 29080668 PMC5927845

[B65] McCance-Katz E. Carroll K. Rounsaville B. (1999). Gender differences in treatment-seeking cocaine abusers–implications for treatment and prognosis. *Am. J. Addict.* 8 300–311. 10.1080/105504999305703 10598213

[B66] McClure E. A. Gipson C. D. Malcolm R. J. Kalivas P. W. Gray K. M. (2014). Potential role of N-acetylcysteine in the management of substance use disorders. *CNS Drugs* 28 95–106. 10.1007/s40263-014-0142-x 24442756 PMC4009342

[B67] Mishra D. Pena-Bravo J. Leong K. Lavin A. Reichel C. (2017). Methamphetamine self-administration modulates glutamate neurophysiology. *Brain Struct. Funct.* 222 2031–2039. 10.1007/s00429-016-1322-x 27709300 PMC5380608

[B68] Nicolas C. Russell T. Pierce A. Maldera S. Holley A. You Z. (2019). Incubation of cocaine-craving after intermittent-access self-administration: Sex differences and estrous cycle. *Biol. Psychiatry* 85 915–924. 10.1016/j.biopsych.2019.01.015 30846301 PMC6534474

[B69] Nicolas C. Zlebnik N. Farokhnia M. Leggio L. Ikemoto S. Shaham Y. (2022). Sex differences in opioid and psychostimulant craving and relapse: A critical review. *Pharmacol. Rev.* 74 119–140. 10.1124/pharmrev.121.000367 34987089 PMC11060335

[B70] O’Brien M. Anthony J. (2005). Risk of becoming cocaine dependent: Epidemiological estimates for the United States, 2000-2001. *Neuropsychopharmacology* 30 1006–1018. 10.1038/sj.npp.1300681 15785780

[B71] Origer A. Le Bihan E. Baumann M. (2014). Social and economic inequalities in fatal opioid and cocaine related overdoses in Luxembourg: A case-control study. *Int. J. Drug Pol.* 25 911–915. 10.1016/j.drugpo.2014.05.015 25002330

[B72] Paxinos G. Watson C. (2007). *The rat brain in stereotaxic coordinates*, 6th Edn. Amsterdam: Elsevier. 10.1016/0165-0270(80)90021-7 6110810

[B73] Peltier M. Sofuoglu M. Petrakis I. Stefanovics E. Rosenheck R. (2021). Sex differences in opioid use disorder prevalence and multimorbidity nationally in the veterans health administration. *J. Dual Diagn.* 17 124–134. 10.1080/15504263.2021.1904162 33982642 PMC8887838

[B74] Pena-Bravo J. Penrod R. Reichel C. Lavin A. (2019). Methamphetamine self-administration elicits sex-related changes in postsynaptic glutamate transmission in the prefrontal cortex. *eNeuro* 6. 10.1523/ENEURO.0401-18.2018 30693312 PMC6348447

[B75] Perry A. Westenbroek C. Becker J. (2013). The development of a preference for cocaine over food identifies individual rats with addiction-like behaviors. *PLoS One* 8:e79465. 10.1371/journal.pone.0079465 24260227 PMC3832528

[B76] Perry A. Westenbroek C. Jagannathan L. Becker J. (2015). The roles of dopamine and α1-adrenergic receptors in cocaine preferences in female and male rats. *Neuropsychopharmacology* 40 2696–2704. 10.1038/npp.2015.116 25900120 PMC4864645

[B77] Peterson A. Hivick D. Lynch W. (2014). Dose-dependent effectiveness of wheel running to attenuate cocaine-seeking: Impact of sex and estrous cycle in rats. *Psychopharmacology* 231 2661–2670. 10.1007/s00213-014-3437-1 24464528

[B78] Peterson B. Mermelstein P. Meisel R. (2014). Estradiol mediates dendritic spine plasticity in the nucleus accumbens core through activation of mGluR5. *Brain Struct. Funct.* 220 2415–2422. 10.1007/s00429-014-0794-9 24878822 PMC5221506

[B79] Potenza M. Hong K. Lacadie C. Fulbright R. Tuit K. Sinha R. (2012). Neural correlates of stress-induced and cue-induced drug craving: Influences of sex and cocaine dependence. *Am. J. Psychiatry* 169 406–414. 10.1176/appi.ajp.2011.11020289 22294257 PMC3690485

[B80] Ramôa C. Doyle S. Lycas M. Chernau A. Lynch W. (2014). Diminished role of dopamine D1-receptor signaling with the development of an addicted phenotype in rats. *Biol. Psychiatry* 76 8–14. 10.1016/j.biopsych.2013.09.028 24199666 PMC3976474

[B81] Ramôa C. Doyle S. Naim D. Lynch W. (2013). Estradiol as a mechanism for sex differences in the development of an addicted phenotype following extended access cocaine self-administration. *Neuropsychopharmacology* 38 1698–1705. 10.1038/npp.2013.68 23481437 PMC3717535

[B82] Rebec G. Sun W. (2005). Neuronal substrates of relapse to cocaine-seeking behavior: Role of prefrontal cortex. *J. Exp. Anal. Behav.* 84 653–666. 10.1901/jeab.2005.105-04 16596984 PMC1389785

[B83] Roth M. Carroll M. (2004). Sex differences in the escalation of intravenous cocaine intake following long- or short-access to cocaine self-administration. *Pharmacol. Biochem. Behav.* 78 199–207. 10.1016/j.pbb.2004.03.018 15219759

[B84] Roura-Martínez D. Diaz-Bejarano P. Ucha M. Paiva R. Ambrosio E. Higuera-Matas A. (2020). Comparative analysis of the modulation of perineuronal nets in the prefrontal cortex of rats during protracted withdrawal from cocaine, heroin and sucrose self-administration. *Neuropharmacology* 180:108290. 10.1016/j.neuropharm.2020.108290 32888961

[B85] Sanchez V. Moore C. Brunzell D. Lynch W. (2014). Sex differences in the effect of wheel running on subsequent nicotine-seeking in a rat adolescent-onset self-administration model. *Psychopharmacology* 231 1753–1762. 10.1007/s00213-013-3359-3 24271035 PMC3969388

[B86] Siemsen B. Giannotti G. McFadden J. Scofield M. McGinty J. (2019). Biphasic effect of abstinence duration following cocaine self-administration on spine morphology and plasticity-related proteins in prelimbic cortical neurons projecting to the nucleus accumbens core. *Brain Struct. Funct.* 224 741–758. 10.1007/s00429-018-1805-z 30498893 PMC6438710

[B87] Smith M. Fronk G. Abel J. Lacy R. Bills S. Lynch W. (2018). Resistance exercise decreases heroin self-administration and alters gene expression in the nucleus accumbens of heroin-exposed rats. *Psychopharmacology* 235 1245–1255. 10.1007/s00213-018-4840-9 29396617 PMC5871570

[B88] Smith M. Walker K. Cole K. Lang K. (2011). The effects of aerobic exercise on cocaine self-administration in male and female rats. *Psychopharmacology* 218 357–369. 10.1007/s00213-011-2321-5 21567123 PMC3752981

[B89] Sofuoglu M. Dudish-Poulsen S. Nelson D. Pentel P. Hatsukami D. (1999). Sex and menstrual cycle differences in the subjective effects from smoked cocaine in humans. *Exp. Clin. Psychopharmacol.* 7 274–283. 10.1037/1064-1297.7.3.274 10472516

[B90] Substance Abuse and Mental Health Services Administration [SAMHSA], (2020). *Detailed tables: Results from the 2020 national survey on drug use.* Rockville, MD: Substance Abuse and Mental Health Services Administration.

[B91] Sun W. Shchepkin D. Kalachev L. Kavanaugh M. (2014). Glutamate transporter control of ambient glutamate levels. *Neurochem. Int.* 73 146–151. 10.1016/j.neuint.2014.04.007 24768447

[B92] Szumlinski K. Shin C. (2018). Kinase interest you in treating incubated cocaine-craving? A hypothetical model for treatment intervention during protracted withdrawal from cocaine. *Genes Brain Behav.* 17:e12440. 10.1111/gbb.12440 29152855 PMC6371797

[B93] Szumlinski K. Wroten M. Miller B. Sacramento A. Cohen M. Ben-Shahar O. (2016). Cocaine self-administration elevates GluN2B within dmPFC mediating heightened cue-elicited operant responding. *J. Drug Abuse* 2:22. 10.21767/2471-853X.100022 27478879 PMC4962921

[B94] Tavares H. Martins S. Lob D. (2003). Factors at play in faster progression for female pathological gamblers: An exploratory analysis. *J. Clin. Psychiatry* 64 433–438. 10.4088/JCP.v64n0413 12716246

[B95] Towers E. Bakhti-Suroosh A. Lynch W. (2021). Females develop features of an addiction-like phenotype sooner during withdrawal than males. *Psychopharmacology (Berl)* 238 2213–2224. 10.1007/s00213-021-05846-3 33907871 PMC8295229

[B96] White K. Brady K. Sonne S. (1996). Gender differences in patterns of cocaine use. *Am. J. Addict.* 5 259–261. 10.1111/j.1521-0391.1996.tb00309.x

