# Supplementary material for: Sex differences in the neuroadaptations associated with incubated cocaine-craving: A focus on the dorsomedial prefrontal cortex
Source: Front Behav Neurosci. 2023 Jan 5;16:1027310. doi: 10.3389/fnbeh.2022.1027310 (PMC9854116; doi:10.3389/fnbeh.2022.1027310)
Supplement: Supplementary file 1 [file Data_Sheet_1.docx]

**Sex Differences in the Neuroadaptations Associated with Incubated Cocaine-Craving: A Focus on the Dorsomedial Prefrontal Cortex**

***Supplemental Material***

**Table of Contacts**

Supplemental Table 1… 2

Supplemental Figure 1 …3

**Table 1. Rat RT-qPCR Primers**

| **Gene ID** | **Gene Name** | **Forward primer nucleotide sequence (5'-3')** | **Reverse primer nucleotide sequence (5'-3')** | **Reference** |
| --- | --- | --- | --- | --- |
| **Gapdh** | Glyceraldehyde-3-phosphate dehydrogenase | GTGGACCTCATGGCCTACAT | TGTGAGGGAGATGCTCAGTG | Koo *et al.* 2015 |
| **B2m** | Beta-2 microglobulin | CGAGACCGATGTATATGCTTGC | GTCCAGATGATTCAGAGCTCCA | Walder *et al* 2014 |
| **Bdnf-IV** | Brain-Derived Neurotrophic Factor exon IV | TTCCACTATCAATAATTTAACTTCTTTGC | CTCTTACTATATATTTCCCCTTCTCTTCAGT | Schmidt *et al.* 2012 |
| **Grin1** | Glutamate Receptor, Ionotropic, N-Methyl D-Aspartate 1 | CACAGGAGCGGGTAAACAACA | TGAGTAGCTCGCCCATCATTC | Wingo *et al.* 2016 |
| **Grin2a** | Glutamate Receptor, Ionotropic, N-Methyl D-Aspartate 2a | GCATCTGCCACAACGAGAAG | CCCGCCATGTTATCGATGTC | Wingo *et al.* 2016 |
| **Grin2b** | Glutamate Receptor, Ionotropic, N-Methyl D-Aspartate 2b | CTGTCCGCCTAGAGGTTTGG | TGCGCTGGGCTTCATCTT | Wingo *et al.* 2016 |

**Table References**

Koo JW, Mazei-Robison MS, LaPlant Q, Egervari G, Braunscheidel KM, Adank DN, Ferguson D, Feng J, Sun H, Scobie KN, Damez-Werno DM, Ribeiro E, Peña CJ, Walker D, Bagot RC, Cahill ME, Anderson SA, Labonté B, Hodes GE, Browne H, Chadwick B, Robison AJ, Vialou VF, Dias C, Lorsch Z, Mouzon E, Lobo MK, Dietz DM, Russo SJ, Neve RL, Hurd YL, Nestler EJ (2015) Epigenetic basis of opiate suppression of Bdnf gene expression in the ventral tegmental area. Nature Neurosci 18:415-422.

Schmidt HD, Sangrey GR, Darnell SB, Schassburger RL, Cha JH, Pierce RC, Sadri-Vakili G (2012) Increased brain-derived neurotrophic factor (BDNF) expression in the ventral tegmental area during cocaine abstinence is associated with increased histone acetylation at BDNF exon I-containing promoters. J Neurochem 120:202-209.

Walder RY, Wattiez AS, White SR, Marquez de Prado B, Hamity MV, Hammond DL (2014) Validation of four reference genes for quantitative mRNA expression studies in a rat model of inflammatory injury. Mol Pain 10:55-59.

Wingo T, Nesil T, Chang SL, Li MD (2016) Interactive effects of ethanol and hiv-1 proteins on novelty-seeking behaviors and addiction-related gene expression. Alcohol Clin Exp Res 40:2102-2113.


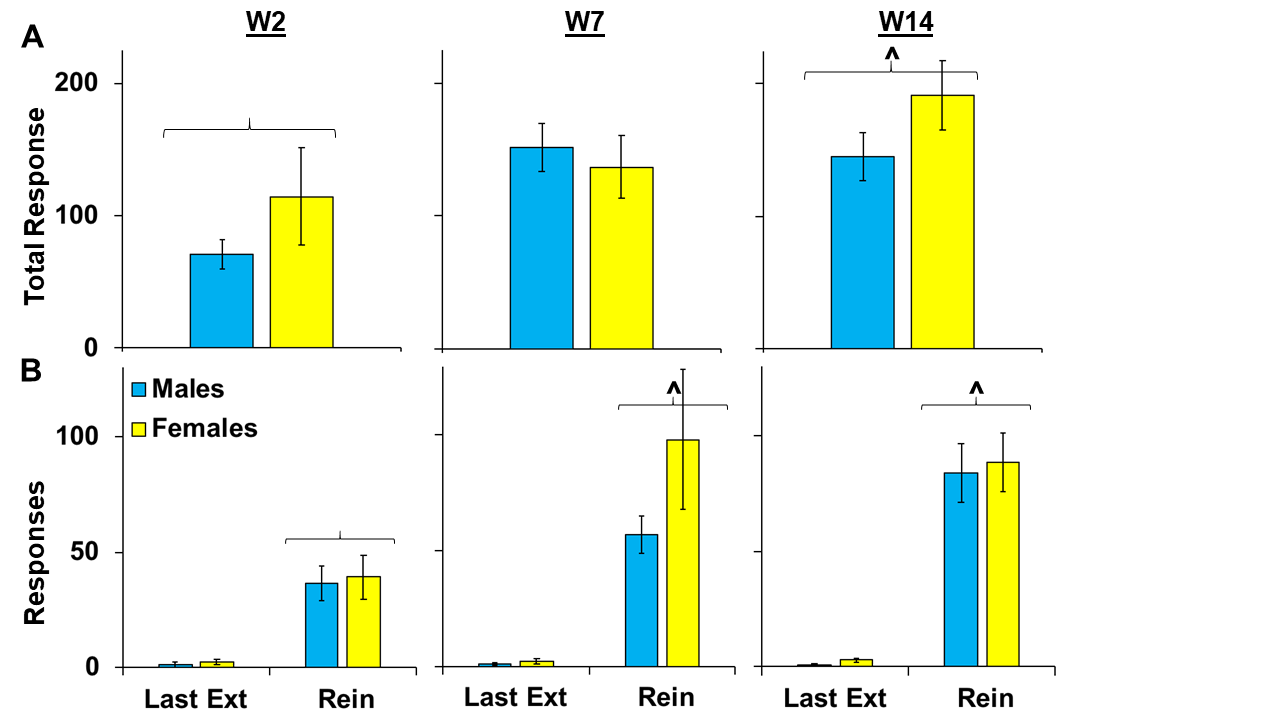


**Supplemental Figure. 1** Withdrawal-specific changes in cocaine craving. Mean (±SEM) number of responses on the lever formerly associated with cocaine across all extinction sessions completed (**A**) and during the last extinction session versus the reinstatement session (**B**) in females and males in the early (W2; n= 13 females,10 males), intermediate (W7; n= 13 females,10 males), and late (W14; n= 13 females,10 males) withdrawal groups. ^Significant difference from early withdrawal group.
